# PReS-FINAL-2316: Churg-Strauss syndrome in a 17-year-old girl

**DOI:** 10.1186/1546-0096-11-S2-P306

**Published:** 2013-12-05

**Authors:** G Ristic

**Affiliations:** 1 Pediatric Immunology and Rheumatology, Mother & Child Health Care Institute of Serbia, Belgrade, Serbia

## Introduction

Churg-Strauss syndrome is a systemic vasculitis accompanied by asthma, eosinophilia and involvement of various organs. It is generally considered a disease of adults with infrequent occurrence in children. We report a patient with Churg-Strauss syndrome manifesting with prominent cardiac and pulmonary involvement.

A 17-year-old girl with previous history of asthma, sinusitis and anorexia nervosa. First time admitted to our Institute with a suspicion of infective endocarditis after surgical correction of scoliosis. Two years later, she was admitted for the second time due to dyspnea and hemoptysis with radiographic patchy infiltrates and a peripheral pulmonary nodule complicated by a pleural and pericardial effusions. She also presented, for the first time, purpura and papulonodular skin lesions on both legs and arms.

## Objectives

To present clinical and laboratory investigations in Churg-Strauss syndrome in childhood.

## Methods

Routine laboratory tests including serum autoantibody screen, lung CT scan and histopathologic examination of skin.

## Results

Laboratory analyses showed an increased WBC count 23.3 × 10^9^/L with predominance of eosinophils (22.1%), normocytic anemia (hemoglobin, 11.2 g/dL), slightly elevated ESR 45 mm/h and C-reactive protein 39.8 mg/L. Serum immunoglobulin were IgA 5.25, IgM 1.78, IgG 20.0 g/l; IgE 3000 IU/ml (normal up to 60 IU/ml). Autoantibody screening was negative, ANCA were negative. Serologic tests to hepatitis B and C, HIV were negative. Serum ACE was normal. Pulmonary CT scan findings were consolidation with nodular lesions. Echocardiographic findings were mitral valve insufficiency with pericardial effusion. Electroneurography showed peripheral sensory neuropathy, while the histopathologic evaluation of skin lesions showed leukocytoclastic vasculitis.

## Conclusion

As the corticosteroid treatment was started, the remission was accomplished in the following months. Our patient illustrates many of the typical features of Churg-Strauss syndrome. Even though, this syndome is rare in pediatric patients it is important to heighten awareness that this serious disease may affect the pediatric population.

## Disclosure of interest

None declared.

